# Inhibition of TGF-β signaling enhances osteogenic potential of iPSC-derived MSCs

**DOI:** 10.1038/s41598-025-89370-w

**Published:** 2025-03-06

**Authors:** Felix Umrath, Sarah-Lena Frick, Valerie Wendt, Andreas Naros, Rüdiger Zimmerer, Dorothea Alexander

**Affiliations:** 1 Department of Oral and Maxillofacial Surgery, University Hospital Tübingen, Osianderstr. 2-8, Tübingen, 72076 Germany; 2 Department of Orthopedic Surgery, University Hospital Tübingen, Tübingen, Germany

**Keywords:** Induced pluripotent stem cells (iPSCs), Mesenchymal stem cells (MSCs), iPSC-derived mesenchymal stem cells (iMSCs), SB431542, TGF-β inhibitor, Osteogenic differentiation, Stem cells, Mesenchymal stem cells, Pluripotent stem cells, Stem-cell differentiation

## Abstract

Mesenchymal stem cells (MSCs) represent the most commonly utilized type of stem cell in clinical applications. However, variability in quality and quantity between different tissue sources and donors presents a significant challenge to their use. Induced pluripotent stem cells (iPSCs) are a promising and abundant alternative source of MSCs, offering a potential solution to the limitations of adult MSCs. Nevertheless, a standardized protocol for the differentiation of iPSCs into iPSC-derived mesenchymal stem cells (iMSCs) has yet to be established, as the existing methods vary significantly in terms of complexity, duration, and outcome. Many straightforward methods induce differentiation by culturing iPSCs in MSC media which are supplemented with fetal bovine serum (FBS) or human platelet lysate (hPL), followed by selection of MSC-like cells by passaging. However, in our hands, this approach yielded inconsistent quality of iMSCs, particularly in terms of osteogenic potential and premature senescence. This study examines the impact of the selective TGF-β inhibitor SB431542 on iMSC differentiation, demonstrating that TGF-β inhibition enhances osteogenic potential and reduces premature senescence. Additionally, we present a reliable, xeno-free method for producing high-quality iMSCs that can be adapted for Good Manufacturing Practice (GMP) compliance, thus enhancing the potential for clinical applications.

## Introduction

Mesenchymal stem cells (MSCs) are regarded as a highly promising therapeutic option in the field of regenerative medicine due to their capacity to differentiate into various cell types, modulate immune responses, and promote tissue repair^1,2^. They are currently under investigation or in clinical use for the treatment of a diverse range of bone and cartilage disorders, cardiac diseases, autoimmune disorders, wound healing, neurological disorders, and graft-versus-host disease (GvHD)^3–9^. In our research on bone regeneration, we utilize, mesenchymal stem cells derived from jaw periosteum (JPCs). Due to their origin, JPCs are cells with a high osteogenic differentiation potential, exhibiting all characteristics of mesenchymal stem cells, including surface marker expression and trilineage differentiation^10^. Consequently, they represent a valuable cellular source for bone regeneration, especially in oral andmaxillofacial surgery.

Despite the promising results observed in preclinical studies, the clinical application of MSCs is currently limited by several unresolved challenges. These include variability in donor-derived MSCs, senescence upon extensive expansion during in vitro cultivation, potential immune rejection in allogeneic settings, and ethical concerns related to tissue sourcing^11–14^.

One strategy to address these challenges is the use of induced pluripotent stem cell-derived mesenchymal stem cells (iMSCs)^15^. iMSCs offer several advantages over primary MSCs, including standardized generation, reduced donor variability and potential rejuvenation of cells from aged donors^16,17^. Furthermore, iPSCs can be expanded indefinitely, thereby providing a virtually limitless supply of cells^18^. iMSCs are derived from patient-specific iPSCs, which can be used to generate autologous iMSCs, thus minimizing concerns regarding immune rejection. Moreover, iPSCs can be derived from readily accessible tissues, such as skin or blood, thus circumventing the ethical concerns associated with bone marrow or adipose tissue donation^19,20^.

The initial development of methodologies for the derivation of iMSCs was conducted within the context of generating MSC-like cells from embryonic stem cells (ESCs)^21–24^. Subsequently, these methods were applied to iPSCs, where they have since undergone further development. The generation of iMSCs is typically achieved through the induction of differentiation through specific protocols involving the use of growth factors, small molecules, and culture conditions that mimic the developmental cues guiding MSC differentiation in vivo. The differentiation process aims to direct iPSCs towards a mesodermal lineage and to promote MSC commitment, which is characterized by a number of typical surface markers (e.g., CD73, CD90, CD105) and functional properties (tri-lineage differentiation potential into osteoblasts, chondrocytes, and adipocytes)^25^.

A variety of differentiation strategies can be employed for the generation of iMSCs. A frequently used approach is the use of 3D culture, which attempts to mimic the physiological microenvironment. This can be achieved through the formation of embryoid bodies, which facilitate cell-cell interactions and paracrine signaling, which is essential for guiding iPSCs towards the MSC fate^26^.

Another common approach is the sequential application of growth factors. iPSCs are exposed to a series of growth factors and cytokines that sequentially drive differentiation towards mesodermal progenitors and eventually MSCs^27^. For example, BMP-4 (Bone Morphogenetic Protein 4) and Activin A are frequently used to initiate mesodermal differentiation, followed by specific cocktails of growth factors to promote MSC specification.

Instead of growth factors, chemical compounds can be used to modulate signaling pathways involved in mesodermal and MSC differentiation^28^. Small molecules targeting pathways like Wnt, Notch, and TGF-β signaling pathways can enhance the efficiency and purity of iMSC generation.

Another relatively straightforward approach to the generation of iMSCs is the cultivation of iPSCs in serum- or thrombocyte lysate-supplemented media^29,30^. The presence of growth factors within these media induces the differentiation of the cells into iMSCs, and subsequently, plastic-adherent cells can be selected through passaging.

In a previous work, we employed this methodology to generate iMSCs^15^. However, the cells exhibited a high expression of senescence markers P16 and P21 as well as senescence associated β-galactosidase (SA-β gal) and low proliferation rates. In addition, the cells showed a high variation in their osteogenic differentiation potential. Consequently, in this study, we attempted to improve the quality of the cells by optimizing the differentiation protocol. Therefore, we tested the effect of SB431542 (SB) as a selective inhibitor of TGF-β signaling on the generation of iMSCs. The TGF-β superfamily, divided into TGF-β, BMPs and activins, plays an important role in cell development and differentiation^31^. These cytokines activate intracellular SMAD-proteins via extracellular receptors, which leads to the transcription of relevant genes^32^. In embryonic stem cells and iPSCs, TGF-β maintains pluripotency by activating SMAD 2/3 signaling pathways and inhibiting SMAD 1/5^33,34^. SB blocks the TGF-β pathway by inhibiting the ALK4, ALK5 and ALK7 receptors, but it does not affect the BMP pathway, thus promoting differentiation^34^. Studies show that SB improves the differentiation efficiency of iMSCs and reduces the formation of senescent cells^35,36^. Here we additionally investigated the effect of SB on the maturation of iMSCs and on the osteogenic differentiation potential of iMSCs.

## Methods

### Cell culture

Jaw periosteal cells (JPCs) derived from three donors were included in this study after obtaining written informed consent. The study was approved by the ethical committee of the University Hospital Tübingen (approval number 618/2017BO2). Jaw periosteal tissue was extracted during routine surgery and JPCs were isolated and expanded as previously reported^37,38^. JPCs and iMSCs were grown in hPL5-medium (DMEM/F12 (Thermo Fisher Scientific Inc., Waltham, MA, USA) + 5% human platelet lysate (hPL, ZKT Tübingen GmbH), 100 U/mL penicillin-streptomycin (Pen-Strep, Lonza, Basel, Switzerland), 2.5 µg/mL amphotericin B (Biochrom, Berlin, Germany)).

iPSCs were cultured on vitronectin (VTN) coated plates and maintained in Essential 8 medium (E8, Thermo Fisher Scientific Inc., Waltham, MA, USA) with daily medium changes and passaged every 4–6 days using 0.5 mM EDTA (Thermo Fisher Scientific Inc., Waltham, MA, USA) and 10 µM Y27632 ROCK inhibitor (Selleck Chemicals LLC, Houston, TX, USA).

### Generation of integration-free iPSCs from JPCs using srRNA

JPCs were reprogrammed to iPSCs using a self-replicating RNA vector as previously published^39^. Briefly, JPCs were incubated in hPL5 medium containing 0.2 µg/mL recombinant B18R protein (eBioscience, San Diego, CA, USA) prior to transfection with a self-replicating VEE-OKSM-GFP RNA. The DNA template of the srRNA used here was a kind gift from S. Dowdy and was modified with an additional GFP gene^40^. From day 1–5 transfected cells were incubated with hPL5 + 25% B18R conditioned medium (BcM) + 1 µg/mL puromycin (Invivogen, Toulouse, France) to select transfected cells. BcM was prepared by transfecting JPCs with B18R-mRNA and collecting the culture medium after 24 h, 48 h and 72 h as described previously^39^. 250 µM histone deacetylase inhibitor sodium butyrate (NaB, Selleck Chemicals LLC, Houston, TX, USA) was added to the medium to enhance reprogramming efficiency. At day 7, the medium was changed to E8 medium (Thermo Fisher Scientific Inc., Waltham, USA) + 25% BcM. After 15–25 days, single iPSC colonies were picked and transferred into VTN-coated separate wells of a 12 well plate containing E8 medium + 10 µM Y27632 ROCK inhibitor (Selleck Chemicals LLC, Houston, TX, USA) and maintained in E8 medium with daily medium changes.

### Differentiation of iPSCs to iMSCs

For iMSC differentiation, iPSCs were grown in 6-well plates until 80% confluency and detached on day 0 using a cell scraper. Cell aggregates were transferred into VTN-coated T75 flasks containing 10 ml of hPL5 medium + 10 µM Y27632. Cells were then designate as iMSCs of passage 0 (P0). On day 1 medium was changed to hPL5 medium either with (+ SB) or without (-SB) 10 µM SB431542 (Selleck Chemicals LLC, Houston, TX, USA). Cells were cultured in P0 until day 10 with medium changes every other day. On day 10 cells were detached using TrypLE Express (Thermo Fisher Scientific Inc., Waltham, USA) and passed through a cell strainer. To determine the percentage of differentiated cells, CD105 expression was measured using flow cytometry. Therefore, 5·10^4^ cells were resuspended in 50 µl FACS-buffer (PBS + 0.1% BSA + 0.1% sodium azide) with 4% Gamunex (Grifols Deutschland GmbH, Frankfurt, Germany) and 5 µl of anti-CD105-APC antibody (BioLegend, San Diego, USA) was added. After 15 min incubation on ice, samples were washed twice and then resuspended in 200 µl FACS-buffer. CD105 expression was measured using a Guava EasyCyte 6HT-2 L instrument (Merck Millipore, Billerica, MA, USA). The percentage of CD105^+^ cells was calculated and used to seed 1·10^6^ CD105^+^ cells into a T75 flask containing 10 ml hPL5. In the following passages, cells were maintained in hPL5 medium with medium changes every 2–3 days and passaged when reaching > 80% confluency.

### Fluorescence microscopy

To monitor differentiating cells, CD105 expression was visualized by immunofluorescence staining. Therefore, cells were fixed with formalin, blocked for 30 min with 2% BSA and incubated over night with a monoclonal mouse anti-CD105 primary antibody (R&D Systems, Minneapolis, USA) at a concentration of 10 µg/ml. The next day, cells were stained with an Alexa-Fluor488 labeled polyclonal goat anti-mouse IgG secondary antibody (Thermo Fisher Scientific Inc., Waltham, MA, USA) at a concentration of 10 µg/ml for 1 h. A sample without primary antibody was used as negative control. F-Actin and nuclei were counterstained with phalloidin-Atto550 (Sigma-Aldrich, St. Louis, USA) and DAPI (BioLegend, San Diego, USA). Images were taken with an AxioObserver.Z1 microscope and AxioVision 4.8.2 software (Carl Zeiss, Oberkochen, Germany).

### Flow cytometric analysis of JPCs, iPSCs, and iMSCs

The expression of pluripotency markers (SSEA-4, TRA-1-60, TRA-1-80) and MSC-markers (CD44, CD73, CD90, CD105) was analyzed by flow cytometry. Cells were detached using TrypLE Express and 1·10^5^ cells per sample were incubated on ice for 15 min in 20 µl blocking buffer (PBS, 0.1% BSA, 0.1 mg/ml sodium azide (Sigma-Aldrich, St. Louis, USA) and 10% Gamunex (human immune globulin solution, Talecris Biotherapeutics GmbH, Frankfurt, Germany)). Then, 50 µl FACS buffer (PBS, 0.1% BSA, 0.1 mg/ml sodium azide) as well as phycoerythrin (PE) and allophycocyanin (APC) conjugated antibodies (see Table 1) were added and incubated on ice for 20 min. After two washing steps with 200 µl FACS buffer, flow cytometry measurements were performed using the Guava EasyCyte 6HT-2 L instrument (Merck Millipore, Billerica, USA).

**Table 1:** List of antibodies (volume per sample, isotype and conjugate) used for flow cytometry.

Human Antigen	Isotype	Conjugate	Company
SSEA4	Human recombinant antibody (REA)	PE	Miltenyi, Bergisch Gladbach, Germany
TRA-1-60		PE	
TRA-1-81		PE	
REA-Isotype		PE	
CD73	Mouse IgG1	PE	BD Biosciences, Franklin Lakes, USA
CD90		PE	
CD105		APC	BioLegend, San Diego, USA
IgG1 - Isotype		APC	
IgG1- Isotype		PE	R&D Systems, Minneapolis, USA

### Osteogenic differentiation

To stimulate osteogenic differentiation, iMSCs were cultivated in osteogenic medium (DMEM/F12 + 10% hPL, 1% Pen-Strep, 1% amphotericin B, 0.1 mM L-ascorbic acid 2-phosphate (Sigma-Aldrich, St. Louis, MO, USA), 10mM βglycerophosphate (AppliChem, Darmstadt, Germany), 4 µM dexamethasone (Sigma-Aldrich, St. Louis, MO, USA)) with medium changes every other day. After 15–25 days, cells were fixed with 4% formalin and monolayers were stained with 1 mL of Alizarin red solution (40 mM, pH 4.2) for 20 min. Unbound dye was washed off with distilled water and images were taken using an inverted microscope (Leica, Wetzlar, Germany). Quantification of calcium phosphate precipitates stained with alizarin red was performed as previously reported^41^. Briefly, bound alizarin dye was solubilized with 10% acetic acid and the absorbance at 405 nm was quantified photometrically after neutralization with 10% NH_4_OH.

### Senescence-associated β-galactosidase (SA-β-gal) activity

To assess the cellular senescence, SA-β-gal activity was detected using a senescence assay kit (Abcam, Cambridge, UK) following the manufacturer’s instructions. Briefly, 2.5·10^4^ cells were seeded in triplicates into 12-well plates containing hPL5. After 48 h, fresh hPL5 medium containing 3 µL/mL of senescence dye was added to the cells and incubated for 1–2 h. After incubation, fluorescence microscopic images were taken using an AxioObserver.Z1 microscope and AxioVision 4.8.2 software (Carl Zeiss, Oberkochen, Germany). Subsequently, cells were detached, pooled, and resuspended in wash buffer (included in the kit) and flow cytometry measurements were performed using the Guava EasyCyte 6HT-2 L flow cytometer (Merck Millipore, Billerica, MA, USA).

### Gene expression analysis of JPCs, iPSCs, and iMSCs

RNA isolation from JPCs and iMSCs was performed using the NucleoSpin RNA kit (Macherey-Nagel, Düren, Germany) following the manufacturer’s instructions. RNA concentration was measured using a Qubit 3.0 fluorometer and the corresponding RNA BR Assay Kit (Thermo Fisher Scientific Inc., Waltham, MA, USA). Total of 0.5 µg of RNA was used for first-strand cDNA synthesis using the SuperScript Vilo Kit (Thermo Fisher Scientific Inc., Waltham, MA, USA). The quantification of mRNA expression levels was performed using the real-time LightCycler System (Roche Diagnostics, Mannheim, Germany). For the PCR reactions, commercial primer kits (Search LC, Heidelberg, Germany), and DNA Master SYBR Green I (Roche, Basel, Switzerland) were used. The amplification was performed with a touchdown PCR protocol of 40 cycles (annealing temperature between 68 and 58 °C), following the manufacturer’s instructions. Copy numbers of each sample were calculated on the basis of a standard curve (standard included in the primer kits), and normalized to the housekeeping gene glyceraldehyde-3-phosphate dehydrogenase (*GAPDH*).

### Statistical analysis

For the evaluation of MSC, iPSC and senescence marker expression, as well as SA-β gal activity, means ± standard deviations were calculated and compared by one-way ANOVA (*p* adjusted using Tuckey’s multiple comparison test).

Alizarin quantification and expression of osteogenic marker genes was evaluated by calculating means ± standard deviations and compared using two-way ANOVA (*p* adjusted using Tuckey’s multiple comparison test). Calculations were performed using GraphPad Prism 10.1.1 software.

## Results

### iMSC differentiation

**Fig. 1:**
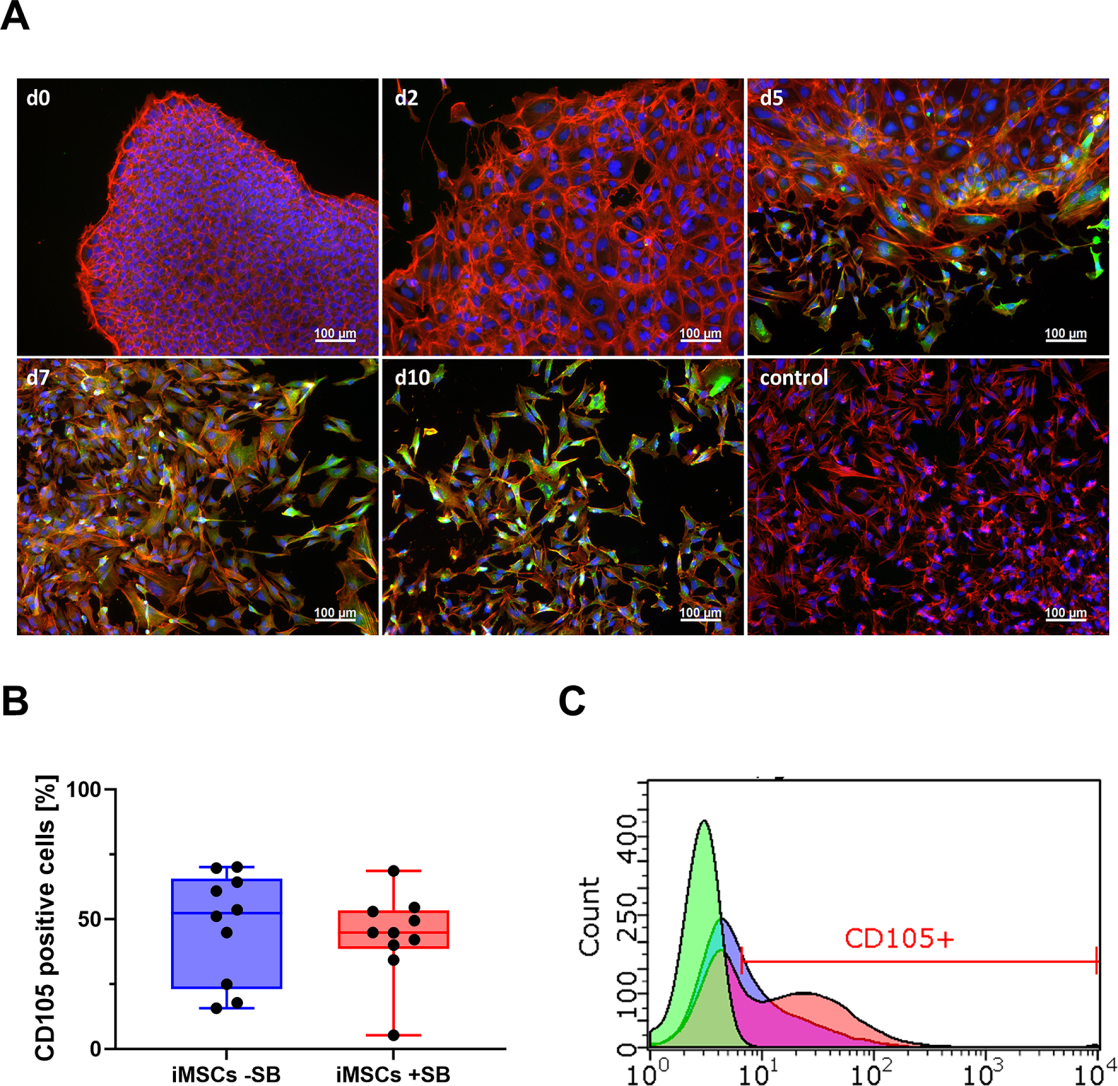
(**A**) Cell morphology during differentiation of iPSCs to iMSCs with SB431542 (+ SB). Representative fluorescence images (10x magnification, scale bar = 100 μm) of undifferentiated iPSCs at day d0 and differentiating cells at different days (d2, d5, d7, and d10) of mesenchymal differentiation (P0). To visualize differentiation, cells were stained for CD105 (green) using a primary mouse anti-CD105 antibody and an AlexaFluor488 labeled secondary goat anti-mouse IgG antibody. The control sample was stained with secondary antibody only. Samples were counterstained with phalloidin-Atto550 (red) and DAPI (blue) to visualize F-actin and nuclei. (**B**) Percentage of CD105^+^ cells at P0 of iMSC differentiation with (+ SB) and without SB431542 (-SB). To determine the percentage of differentiated cells and the resulting cell density after passaging to P1, CD105 expression in iMSCs of passage 0 was determined by flow cytometry. The two groups were compared using a paired Student’s t-test (*n* = 10). (**C**) Representative histogram of CD105 expression in iMSCs + SB (blue), -SB (red) and unstained control (green).

In comparison to previous attempts to generate iMSCs from iPSCs, several modifications were made to the differentiation protocol in order to standardize and enhance differentiation efficiency of resulting iMSCs. Instead of plating iPSCs as a single-cell suspension (previous experiments), cells were gently detached using a cell scraper and then plated in MSC medium. This led to the formation of larger three-dimensional cell aggregates on the cell culture plate, similar to adherent embryoid bodies. From day 1 to day 10, SB431542 (SB) was added to the medium. In this initial phase, distinct zones comprising cells with varying differentiation characteristics emerged around the center of the aggregates. While the center was predominantly composed of iPSC-like cells, an outer zone with spindle-shaped MSC-like cells emerged. Figure 1A shows CD105 expression of differentiating cells during passage 0. It can be observed that spindle-shaped cells expressing CD105 appear at the edges of the colonies (Figs. 1A, d2) and then grow outwards.

After 10 days, the cells were passaged and plated on uncoated cell culture flasks to select iMSCs by plastic adherence. In this step, a critical factor is the cell density after passaging (in P1), which is optimal at 0.5-1·10^6^ cells per T75 flask.

As undifferentiated cells will die after passaging at this step, the cell density in P1 depends on the proportion of differentiated, MSC-like cells. Thus, the expression of CD105 was analyzed by flow cytometry and the percentage of CD105^+^ cells was determined (Fig. 1B + C) to quantify MSC-like cells. As shown in Fig. 1B, no significant differences between differentiation with (+ SB) and without SB (-SB) was detected. However, the percentage of CD105^+^ cells is subject to high fluctuations. With this method, the cell density in P1 was controlled and growth inhibition by too low cell densities could be prevented.

### iMSC characterization

#### iPSC and MSC surface marker expression

**Fig. 2:**
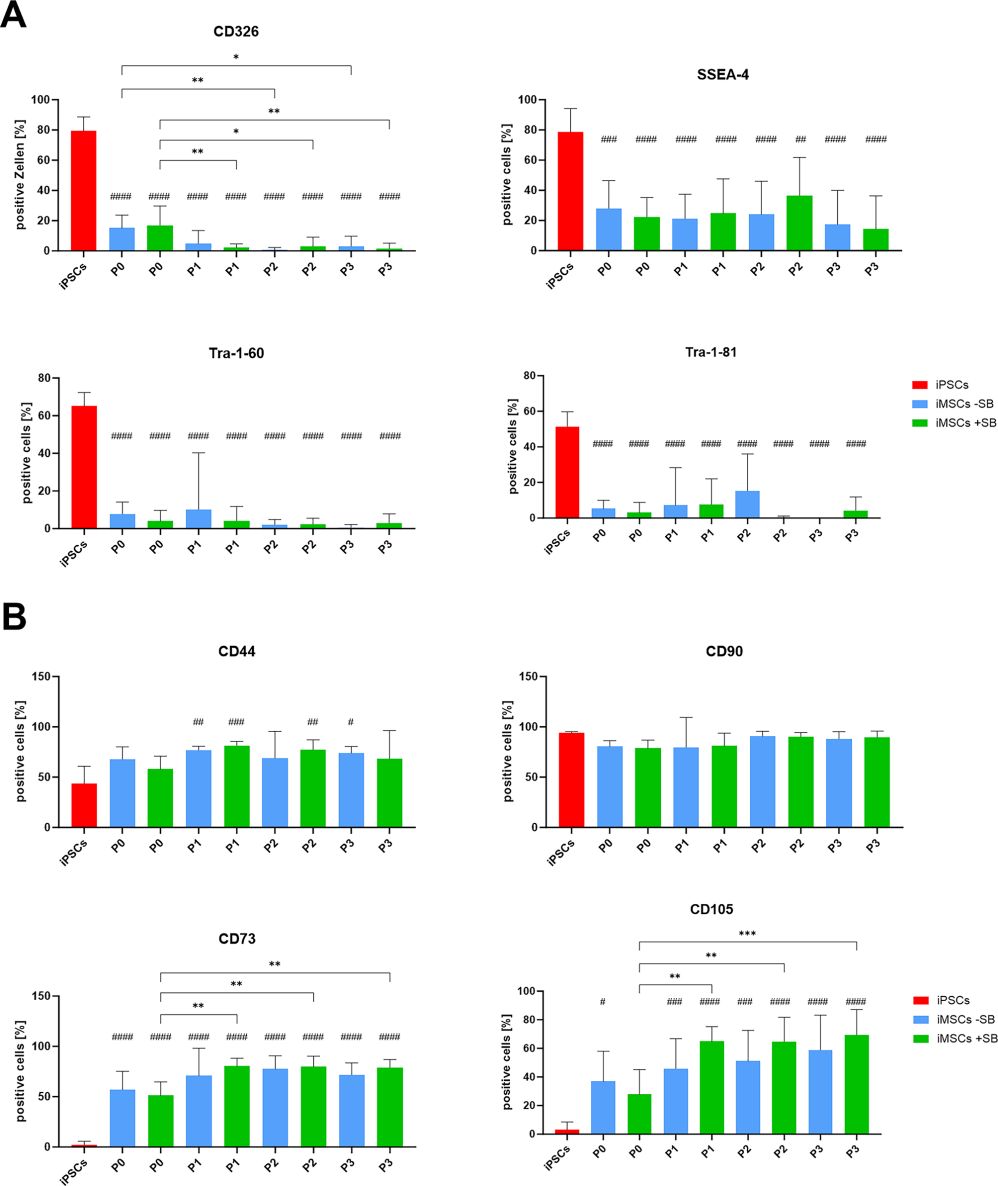
Surface marker expression during iMSC-differentiation. Expression of (**A**) iPSC-markers (CD326, SSEA-4, SSEA-4, Tra-1-60, Tra-1-81) and (**B**) of MSC-markers (CD44, CD73, CD90, CD105) was measured by flow cytometry. Percentages of positive cells from iPSCs (red) and iMSCs differentiated with (green) and without SB (blue) at different passages of the iMSC differentiation process. Means and standard deviations are displayed and were compared by one-way ANOVA and Tuckey’s multiple comparison test (*n* ≥ 7, * *p* < 0.05, ** *p* < 0.01, *** *p* < 0.001, **** *p* < 0.0001; # indicate significant p-values compared to iPSCs).

In the course of the differentiation process, the expression of iPSC (Fig. 2A) and MSC markers (Fig. 2B) was determined when passing to the next passage, using flow cytometry. The objective was to monitor cell maturation and to determine the time period required for this process. Furthermore, the impact of SB on marker expression during differentiation was investigated.

Figure 2A illustrates the expression of iPSC surface markers. CD326 is an epithelial cell marker that was employed in this study for the observation of epithelial-to-mesenchymal transition. The proportion of CD326-positive cells declined significantly during iMSC differentiation with or without SB from 79.4% to values below 5% in P1. No notable differences were observed between the -SB and + SB groups. Analyzed iMSCs of P0 exhibited significantly higher (*p* < 0.05) CD326 percentages compared to later passages, indicating incomplete maturation.

The proportion of SSEA-4-expressing cells declined significantly from 78.6% (iPSCs) to 27.8% (-SB) and 22.4% (+ SB) in iMSCs of P0. The expression decreased further to 17.4% (-SB) and 14.3% (+ SB) in P3. No significant differences were observed between cells differentiated with and without SB or between different passages.

The iPSC markers Tra-1-60 and Tra-1-81 demonstrated a significant decline in the proportion of positive cells in iMSCs of both groups in P0. In P3, both iMSC groups exhibited values below 5% positive cells. Once again, no significant differences were observed between cells differentiated with or without SB, or between different passages.

The percentage of CD44-expressing iMSCs increased significantly during differentiation, rising from 43.7% (iPSCs) to 77.0% in the absence of SB (*p* = 0.0025) and 81.5% in the presence of SB (*p* = 0.0003) in passage 1. Thereafter, the percentage decreased to 74.0% in the absence of SB (*p* = 0.0113) and 68.4% in the presence of SB (ns) in Passage 3. No significant differences were observed between the cells that had been differentiated with or without SB.

CD90 is a characteristic marker of MSC surface expression; however, iPSCs also demonstrated a substantial level of CD90 expression. Consequently, no significant differences could be identified between both groups.

The markers CD73 and CD105 were found to be the most suitable to monitor the differentiation of iMSCs. iPSCs exhibited minimal expression of CD73 (2.1%), which increased significantly during mesenchymal cell maturation and reached a plateau between 71.6% and 80.3% in P1 (+ SB) or P2 (-SB). All values exhibited a significant increase compared to the iPSCs (*p* < 0.0001), yet no significant differences were observed between the -SB and + SB differentiated iMSCs. The + SB differentiated iMSCs in P0 exhibited significantly lower (*p* < 0.01) percentages of CD73^+^ cells compared to those in higher passages, indicating incomplete maturation of the cells.

Similar to CD73, iPSCs also exhibit minimal CD105 expression (3.1%). Similarly, there is a significant increase in the number of positive cells during iMSC differentiation. In the -SB group, the proportion of CD105^+^ cells exhibited a slower rate of increase and reached a maximum value of only 58.8% in P3. In contrast, the level of positive cells in the + SB group already reached 64.9% in P1 and then increased only slightly to 69.4% by P3. The + SB differentiated cells in P0 exhibited significantly lower (*p* < 0.01) amounts of CD105^+^ cells compared to later passages, indicating incomplete maturation of the cells.

#### Expression of iPSC and MSC marker genes

Similar to surface marker expression, gene expression of iPSC and MSC markers was analyzed during the iMSC differentiation process. In addition, the results were compared to the parental JPCs to analyze similarities in gene expression in JPCs and iMSCs.

**Fig. 3:**
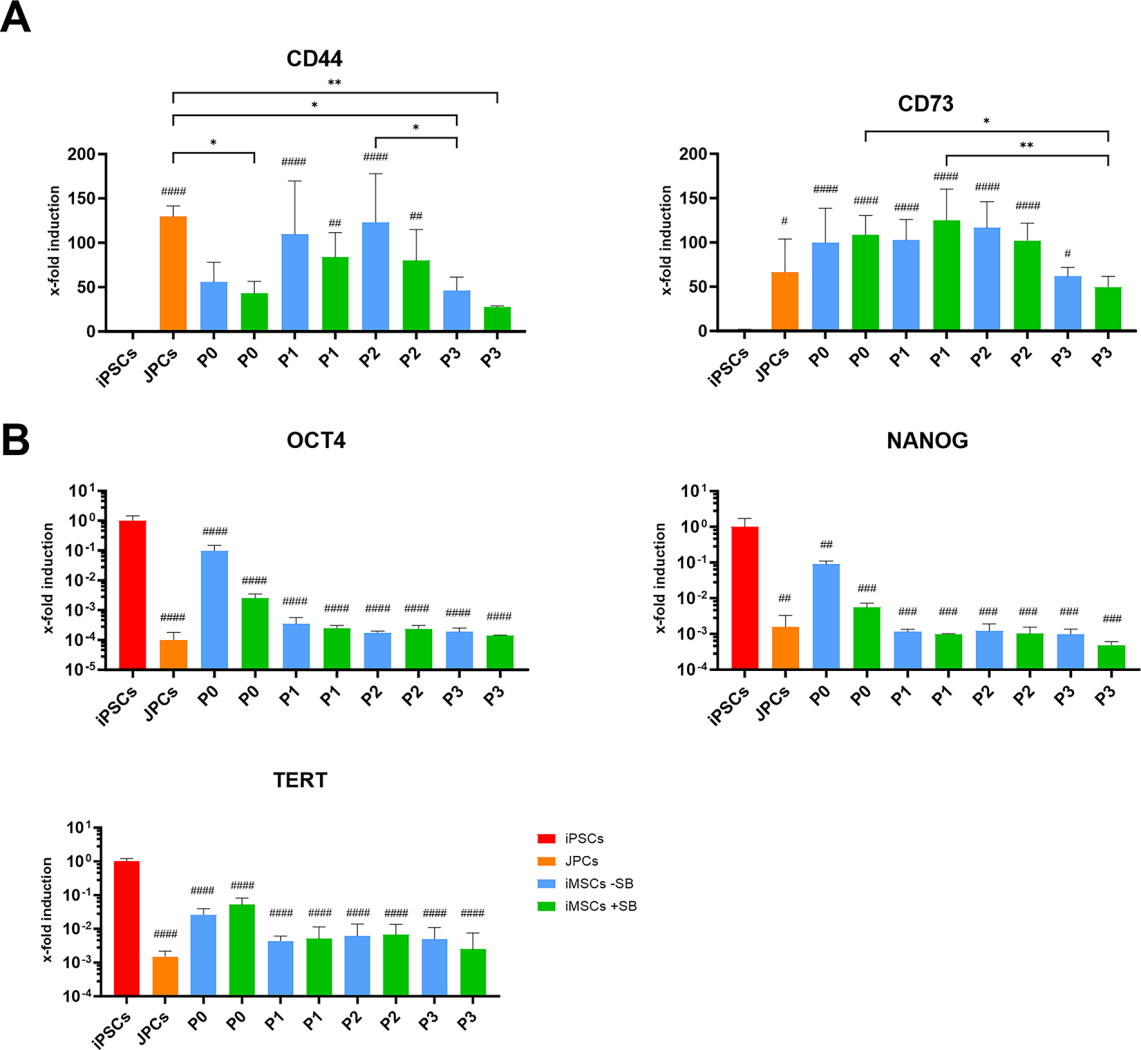
Gene expression of MSC- and iPSC-markers during iMSC-differentiation. Expression of (**A**) MSC-markers (CD44, CD73) and (**B**) of iPSC-markers (OCT4, NANOG, TERT) was measured by qPCR. Absolute mRNA copy numbers of iPSCs (red), JPCs (orange) and iMSCs differentiated with (green) and without SB (blue) at different passages of the differentiation process were normalized to the housekeeping gene glycerinaldehyde-3-phosphate dehydrogenase (GAPDH) and x-fold induction values relative to the iPSC sample were calculated. Means + SD (*n* = 4) are displayed and were compared by one-way ANOVA and Tuckey’s multiple comparison test (* *p* < 0.05, ** *p* < 0.01, *** *p* < 0.001, **** *p* < 0.0001; # indicate significant p-values compared to iPSCs).

CD44 expression levels were found to be lowest in iPSCs, and highest in parental JPCs exhibiting a 129-fold higher expression (*p* < 0.0001) (Fig. 3). The expression of CD44 initially increased during iMSC differentiation and reached a peak in either P1 (+ SB) or P2 (-SB), subsequently decreasing in P3 (-SB; *p* = 0.0287). In P3, CD44 gene expression in iMSCs of the -SB (*p* = 0.0287) and + SB (*p* = 0.0033) groups were found to be significantly lower than those of the JPCs. Significant differences between the -SB and + SB differentiated iMSCs were not observed. However, the -SB differentiated cells exhibited a tendency of higher CD44 expression.

As expected, CD73 expression in iPSCs was found to be low, whereas parental JPCs showed a significantly higher expression (66.5-fold higher, *p* = 0.0135) (Fig. 3). Similar to the expression of CD44, CD73 expression levels reach a maximum in P1 (+ SB, 124.8) and P2 (-SB, 116.6) of iMSC differentiation. In P0, the expression of iMSCs of -SB (99.8) and + SB (108.3) groups is higher than that of JPCs (66.5). After reaching a maximum, the values decline to 61.7 (-SB) and 49.7 (+ SB) in P3, and remain slightly below levels detected in JPCs. No significant differences were observed between the -SB and + SB differentiated iMSCs.

Figure 3B illustrates the expression of the iPSC markers OCT4, NANOG and TERT. For better visualization, the data are presented on a logarithmic scale. While the iPSCs exhibit the highest expression of each of the three iPSC markers, they are significantly downregulated in JPCs. Furthermore, all iMSC samples show a significantly lower expression of the three markers compared to iPSCs. The expression of all iPSC markers is higher in iMSCs at P0 compared to later passages, which suggests that the maturation process is in progress. The only discernible differences between the -SB and + SB group were observed for OCT4 and NANOG in P0. In this passage, the -SB group exhibited clearly higher gene expression than the + SB group. This could indicate an accelerated maturation process in the presence of SB, however, the data is not statistically significant. In P3, the expression levels of iPSC markers in iMSCs differentiated with and without SB were comparable to those observed in JPCs.

### Osteogenic differentiation

**Fig. 4:**
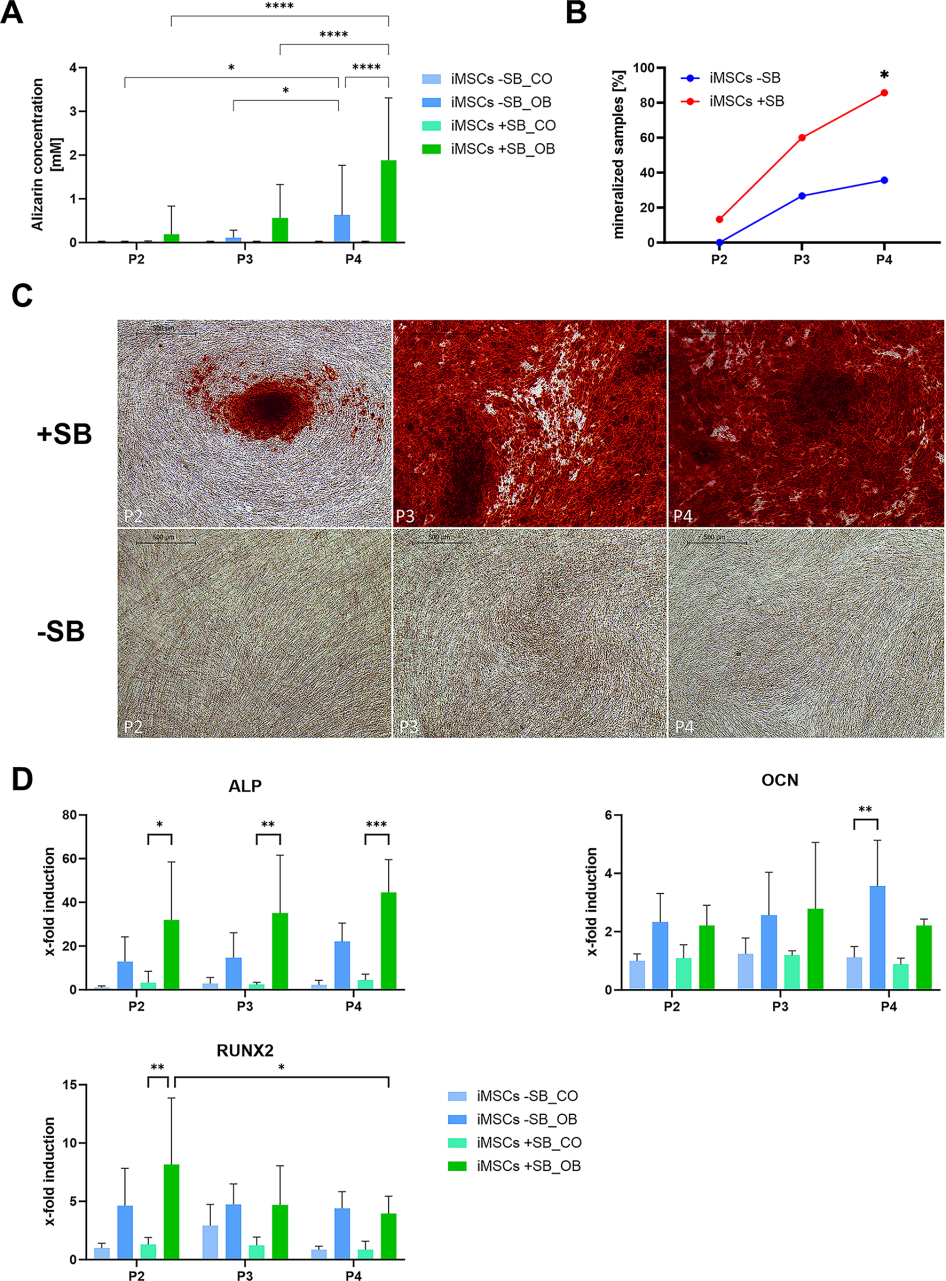
Osteogenic differentiation of iMSCs in consecutive passages. iMSC mineralization of passages 2–4 was visualized by alizarin red staining after 21 days of osteogenic induction. (**A**) Alizarin concentration was quantified photometrically and means + SD are displayed (*n* ≥ 12). (**B**) Increase in mineralization of iMSCs differentiated with and without SB after osteogenic stimulation for 21 days. Percentage of mineralizing samples was determined by counting mineralizing and non-mineralizing wells after alizarin staining. The groups were compared using Fisher’s exact test (* *p* < 0.05). (**C**) Representative images of alizarin staining (4x magnification, scale bar = 500 μm). (**D**) Expression of osteogenic marker genes (ALP, OCN and RUNX2) in iMSCs was quantified by qPCR after 15 days of treatment with control (CO) and osteogenic medium (OB). Absolute mRNA copy numbers were normalized to the housekeeping gene glycerinaldehyde-3-phosphate dehydrogenase (GAPDH) and displayed as x-fold induction values relative to the iMSCs -SB_CO P2 sample (means + SD, *n* = 4). The mean values of gene expression and alizarin quantification were compared by 2-way ANOVA and Tuckey’s multiple comparison test (* *p* < 0.05, ** *p* < 0.01, *** *p* < 0.001, **** *p* < 0.0001).

The capacity for osteogenic differentiation is of major importance for the application of iMSCs in bone regeneration. To determine whether the osteogenic potential enhances during the mesenchymal cell differentiation process, iMSCs were subjected to osteogenic stimulation in P2, P3, and P4. Figure 4A illustrates the degree of cell mineralization, as quantified after alizarin staining. It is evident that the degree of mineralization of the cells increases with the passage number. Both -SB and + SB differentiated iMSCs exhibited a significantly higher degree of mineralization in P4 compared to earlier passages. Furthermore, there was a significant improvement in mineralization in the + SB group. The iMSCs + SB exhibited significantly higher values (*p* < 0.0001) in P4 in comparison to the -SB group.

This observation was also evident in the proportion of mineralizing and non-mineralizing samples (Fig. 4B). Both groups exhibited a clear trend with increasing proportions in higher passages. However, the proportion of the -SB group in P4 was significantly lower with only 35.7%, while in the + SB group, mineralization was detected in 85.7% of the wells (*p* = 0.0183). The overall p-value for all passages is *p* < 0.0001, demonstrating a statistically significant influence of SB on the occurrence of mineralization.

The progression from P2 to P4 and the contrasts between the -SB and + SB groups are illustrated in Fig. 4C by microscopic images of cells from a single donor. The contrasts between the -SB and + SB groups, as well as the increasing mineralization from P2 to P4, are clearly evident through the use of alizarin staining.

Furthermore, the expression of osteogenic marker genes, Alkaline Phosphatase (ALPL), Osteocalcin (OCN), and Runt-Related Transcription Factor 2 (RUNX2) (Fig. 4D), was investigated. The expression of ALPL largely reflects the course of mineralization (Fig. 4A). This demonstrates a consistent increase in ALPL expression from P2 to P4, with a stronger expression in the + SB group. In contrast, the expression of OCN and RUNX2 does not demonstrate clear differences between the two groups. Here, only differences between the control group (CO) and osteogenically differentiated cells are identifiable.

#### Comparison of various SB incubation methods

**Fig. 5:**
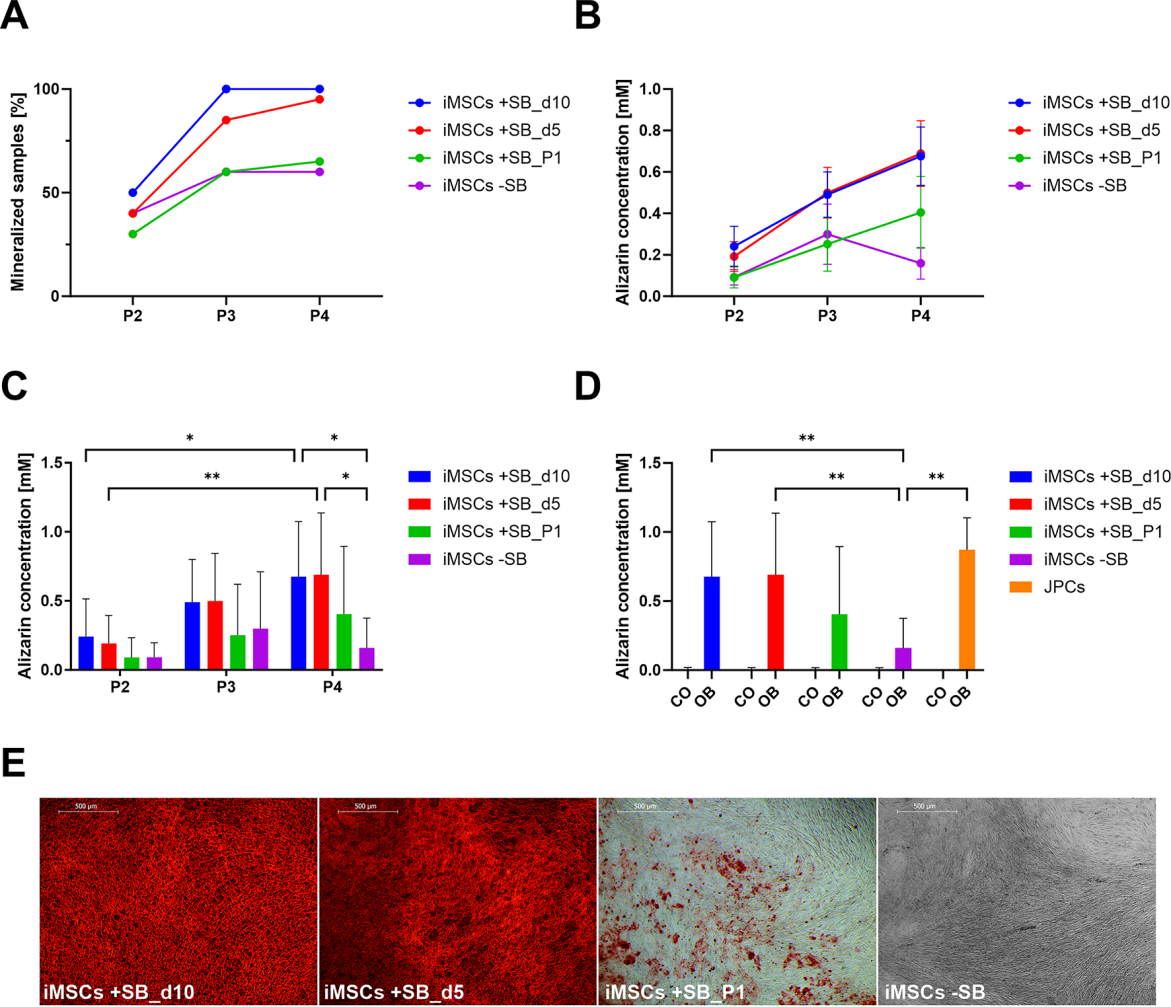
Osteogenic potential of differently generated iMSCs. iMSCs were differentiated using four different protocols (SB_d10 = SB incubation in P0 until d10, SB_d5 = SB incubation in P0 until d5, SB_P1 = SB incubation in P1 for 10 days, -SB = without SB). Differences in mineralization of iMSCs of passages 2–4 was visualized by alizarin red staining after 21 days of osteogenic differentiation. (**A**) Increase in mineralization of differently generated iMSCs of different passages after stimulation with osteogenic medium for 21 days. Percentage of mineralizing samples was determined by counting mineralized and non-mineralized wells after alizarin staining. (**B**) Mean alizarin concentrations and SEM are displayed as connected lines to visualize development of osteogenic potential in different passages. (**C**) Alizarin concentrations of osteogenically differentiated iMSCs were quantified photometrically and means + SD are displayed and compared by 2-way ANOVA and Tuckey’s multiple comparison test. (**D**) Alizarin concentration of iMSCs of passage 4 and originating JPCs treated with osteogenic (OB) and control medium (CO) was quantified photometrically and means + SD are displayed and compared by 2-way ANOVA and Tuckey’s multiple comparison test (*n* = 8, * *p* < 0.05, ** *p* < 0.01). (**E**) Representative images of differently generated iMSCs after osteogenic differentiation in passage 4 and alizarin staining (4x magnification, scale bar = 500 μm).

In separate experiments, the impact of SB on the osteogenic differentiation potential of iMSCs was investigated in more detail. To this end, the duration of SB addition (for 5 days, or for 10 days in P0) and timepoint of application (P0 or P1) were varied. Thus, four groups were tested: SB incubation in P0 until d10 (SB_d10), SB incubation in P0 until d5 (SB_d5), SB incubation in P1 (not in P0) for 10 days (SB_P1), and without SB (-SB). Subsequently, the cells underwent osteogenic differentiation and their mineralization capacity was evaluated. Figure 5A depicts the proportion of mineralized iMSC samples at different passages (P2 to P4). The data demonstrate a clear trend towards increased mineralization at higher passages. The proportion of mineralized wells was found to increase most rapidly in iMSCs incubated with SB in P0 until day 10 (SB_d10), reaching 100% in P3. A somewhat slower increase is observed in iMSCs incubated with SB in P0 until day 5 (SB_d5). In P3, 85% of the cells reach this stage, while in P4, 95% do so. In contrast, only 60% of the osteogenically differentiated iMSCs without SB (-SB) and the iMSCs differentiated in the presence of SB in P1 (SB_P1) demonstrated the capacity for mineralization. Furthermore, the proportion shows either no or only a slight increase in P4 (-SB = 60%, SB_P1 = 65%), indicating that the addition of SB has only a notable impact at the early stages of iMSC differentiation.

Figure 5B illustrates the progression of calcium phosphate precipitates formed from P2 to P4 in differently generated iMSCs, as quantified by photometric measurements of alizarin concentration (mM). While the amounts of calcium phosphate precipitates in the -SB group demonstrate no increase from P2 to P4, all other groups exhibit a clear increase in precipitation. The SB_P1 group exhibits a slight increase (+ 0.314 mM), whereas the SB_d5 (+ 0.497 mM) and SB_d10 (+ 0.434 mM) groups demonstrate a strong increase.

Figure 5C presents the same data, but with the significant differences between the groups highlighted. The groups SB_d10 (*p* = 0.0273) and SB_d5 (*p* = 0.0097) exhibited a significant increase in alizarin concentrations from P2 to P4. Furthermore, these groups exhibited significantly higher values than the -SB group (SB_d10: *p* = 0.0128, SB_d5: *p* = 0.0102) in P4. The SB_P1 group exhibited markedly lower values than the SB_d10 and SB_d5 groups, without reaching statistical significance.

In Fig. 5D, the alizarin concentrations of the differently produced iMSCs in P4 are compared with those of the original JPCs. The data show that calcium concentrations of SB_d10 and SB_d5 groups are close to those detected in JPCs, which are slightly higher. In contrast, the values of the SB_P1 group are markedly lower and concentrations detected in the -SB group are significantly lower (*p* = 0.0031). Representative images of alizarin staining of iMSC groups in P4 are shown in Fig. 5E.

### Expression of senescence markers

**Fig. 6:**
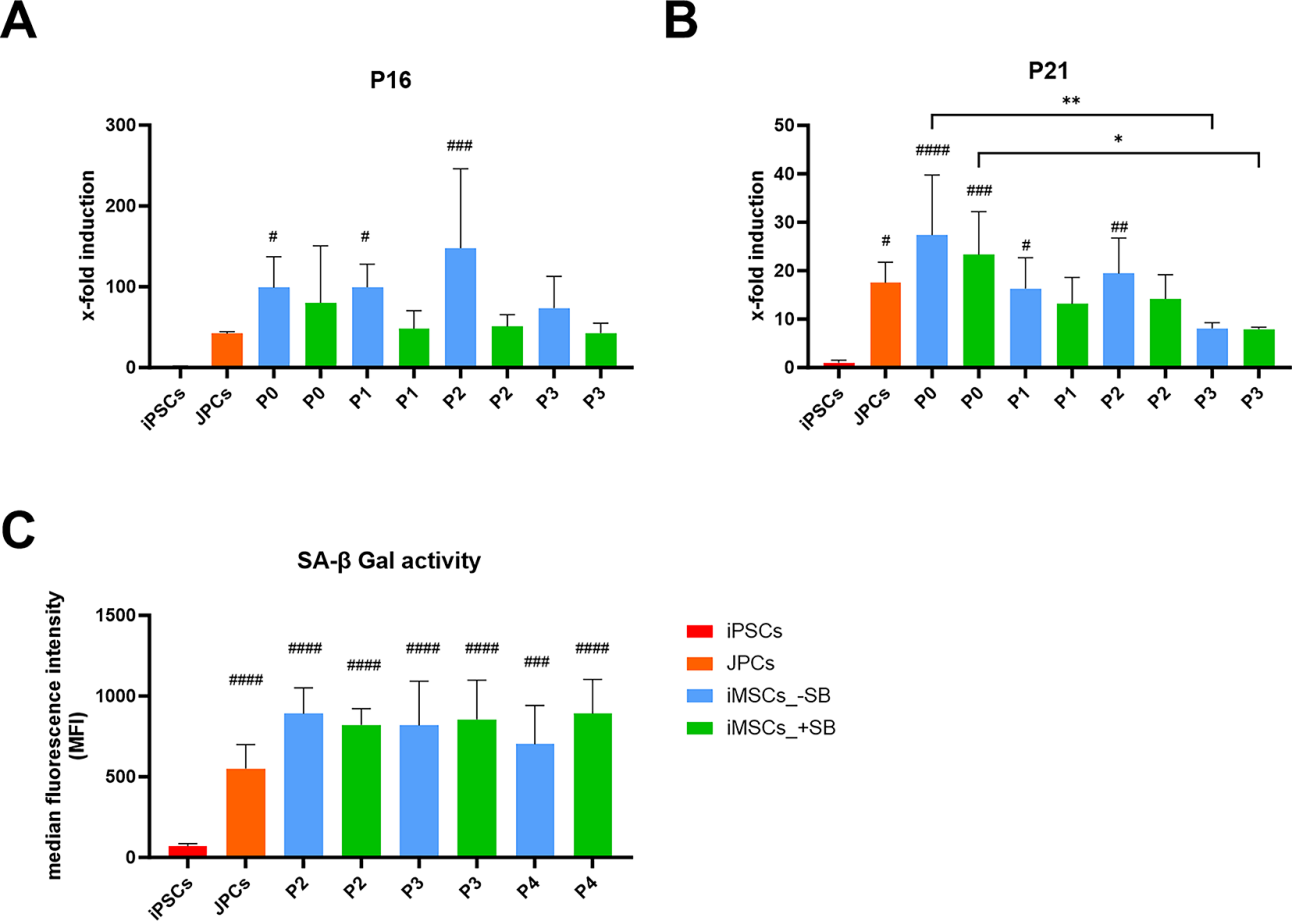
Senescence-associated β-galactosidase (SA-β gal) activity and expression of senescence marker genes during iMSC-differentiation. (**A**) Gene expression of P16 and P21 of iPSCs (red), JPCs (orange) and iMSCs differentiated with (green) and without SB (blue) at different passages of the differentiation process was quantified by qPCR. Absolute mRNA copy numbers were normalized to the housekeeping gene glycerinaldehyde-3-phosphate dehydrogenase (GAPDH) and x-fold induction values were calculated relative to the iPSC’s values. Means + SD (*n* ≥ 3) are displayed. (**B**) Activity of SA-β gal was measured by flow cytometry. Means of median fluorescence index (MFI) values + SD (*n* ≥ 3) are displayed. Gene expression and SA-β gal activity data was analyzed using one-way ANOVA and Tuckey’s multiple comparison test (* *p* < 0.05, ** *p* < 0.01, *** *p* < 0.001, **** *p* < 0.0001, # indicate significant p-values compared to iPSCs).

The expression of senescence markers is indicative of stress during differentiation, manifesting primarily during the isolation of undifferentiated cells. Additionally, the expression of senescence markers is associated with cellular aging. For this reason, the expression of typical senescence-associated genes (P16 and P21, Fig. 6A and B) and the activity of senescence-associated β-galactosidase (SA-β-gal) were analyzed in iMSCs generated with and without SB, across passages 0 to 3. The data were compared with those of iPSCs and the original JPCs (Fig. 6C).

P16 is minimally expressed in iPSCs and exhibits a 41.8-fold higher gene expression in JPCs. iMSCs generated without SB do not demonstrate a clear trend between P0 and P3. Overall, gene expression levels are clearly higher than those of JPCs, ranging from 73.6 to 147.8-fold higher than those of iPSCs. In contrast, iMSCs differentiated with SB initially exhibit a relatively high P16 expression (80-fold higher than iPSCs), followed by a decline to 42.6-fold higher by P3. This level of gene expression is comparable to that detected in the original JPCs.

P21 is also expressed at low levels in iPSCs, while JPCs exhibit a 17.5-fold higher gene expression. iMSCs with and without SB showed a declining trend from P0 to P3. In P0, the P21 expression levels in iMSCs are significantly higher than in P3, with values of 27.3-fold (-SB, *p* = 0.0047) and 23.3-fold higher (+ SB, *p* = 0.0042) than iPSCs. Additionally, the values of iMSCs in P0 are slightly higher than those of JPCs. Conversely, the gene expression in P3 is notably lower than that of JPCs (-SB = 8.1- fold, +SB = 7.9-fold higher than iPSCs).

In addition to measuring the expression of senescence related genes, the activity of senescence-associated β-galactosidase (SA-β-gal) was quantified. Figure 6C illustrates a low level of SA-β-gal activity in iPSCs (MFI 69.8) and markedly higher levels in JPCs (MFI 550.5). The SA-β-gal activity was examined in iMSCs +/-SB from P2 to P4. No notable differences were observed between the two groups or across different time points. However, MFI values of iMSCs were found to be slightly higher than those of JPCs, with values ranging between 702.3 and 821.3.

## Discussion

In this study, we investigated the effect of the specific inhibitor of TGF-β signaling SB431542 (SB) on the differentiation of iMSCs and their osteogenic potential. In addition, the goal was to monitor the maturation of the cells and determine the timing at which the cells are ready for subsequent applications in regenerative medicine. To this end, we examined the expression of surface markers and, we stimulated iMSCs for osteogenic differentiation after each passage and examined the expression of osteogenic markers and the cell mineralization.

iMSCs generated with (+ SB) and without (-SB) SB431542 did not differ significantly in the expression of the iPSC and MSC surface markers analyzed in this study. The MSC markers (CD44, CD73, CD90, CD105) initially increased in P0 and mostly reached a plateau at P1 or P2, whereas the expression of the iPSC markers (CD326, SSEA-4, Tra-1-60, Tra-1-81) decreased and also reached a plateau in P1 in the + SB and -SB group. Considering only surface marker expression would indicate that the mesenchymal differentiation of iMSCs was almost completed at passage 1–2. However, analyses of the osteogenic differentiation of iMSCs shows that the osteogenic potential is not yet fully developed at that stage. The highest mineralization potential was reached in passage 4, and later passages were not tested in this study.

In terms of cell mineralization, significant differences were observed between the iMSC groups differentiated in the presence or absence of SB. The + SB iMSCs exhibited a significantly higher osteogenic potential than the -SB iMSCs. In both groups, the formation of calcium phosphate precipitates, the number of mineralizing samples, and the gene expression of ALPL increased with increasing passage, clearly demonstrating a maturation process. However, in the -SB iMSC group, these values remained at a significantly lower level. As no maximum could be reached during the examined period (passage 2–4), it cannot be definitively stated whether the osteogenic potential of the -SB iMSCs is overall lower and will remain at that level, or whether the maturation of the cells is merely slower compared to the + SB group. From the perspective of potential clinical applications, a rapid maturation process is advantageous. A permanently lower osteogenic potential of the -SB iMSCs could indicate that at least a part of these cells was differentiating into a cell type with a similar surface marker profile but without exhibiting osteogenic differentiation capacity, such as fibroblasts. Further analysis of additional surface markers, such as MSCA-1, would be beneficial in order to directly assess the stem cell potential of resulting cells and enable cell sorting^41^.

To gain further insight into the effect of TGF-β inhibition by SB, the incubation period and time point was varied (Fig. 5). The results demonstrate that SB exerts its primary effect during the initial five days of differentiation. No significant differences were observed between the incubation periods until d5 and until d10. The impact of SB supplementation in passage 1 was markedly diminished even when the incubation period was extended, in comparison to the administration of SB in passage 0 until day 5. It can therefore be assumed that SB acts at an early stage of differentiation, influencing the differentiation into a specific lineage. As demonstrated by Loh and co-authors, the inhibition of individual signaling pathways, including the TGF-β pathway, can be a determining factor in the early mesodermal development, influencing the differentiation of cells towards specific lineages, such as bone, skeletal muscle, fibroblasts, or cardiomyocytes and preventing the differentiation towards the ectodermal and endodermal lineages^27^. The observed minor positive effect of SB supplementation in P1 is likely attributed to the presence of undifferentiated cells in P1.

While numerous protocols for the generation of iMSCs have been developed, a universally accepted standard is still to be established. The differentiation of iPSCs through the switch from iPSC medium to serum-containing MSC medium is an attractive method due to its simplicity^42,43^. However, this approach yielded unsatisfactory and inconsistent results in our laboratory.

The previously generated iMSCs exhibited reduced proliferation rates and mitochondrial activity compared to original JPCs^15^. Additionally, they exhibited elevated expression levels of the senescence markers P16 and P21, as well as increased SA-β-Gal activity^15^. However, replicative senescence could be excluded based on the observation of elongated telomeres^15^. We therefore assumed that the iPSC handling was not optimal and resulted in stress-induced senescence.

It is well documented, that the dissociation of iPSCs without the use of a Rho-associated protein kinase (ROCK) inhibitor results in apoptosis^44–47^. Although the ROCK inhibitor prevents apoptosis, dissociation of iPSCs causes cellular stress, which may induce senescence^44^. Stress-induced reactive oxygen species (ROS) production and the activation of the P16 pathway, triggered by the disruption of iPSC colonies, may underlie this senescence phenotype, analogous to tissue damage-induced senescence^48,49^. Gokoh et al. demonstrated that by avoiding cellular stress and employing an optimized protocol, it is possible to mitigate senescence in iPSC-derived hemangioblast cells^50^. It can thus be postulated that cellular stress during the differentiation process similarly induces senescence in iMSCs. Accordingly, in our study, iPSCs were detached using a cell scraper to initiate differentiation and reduce cellular stress, without further dissociation. The methodology demonstrated reliable attachment of the cell conglomerates, which exhibited outgrowth of spindle-shaped MSC-like cells within 10 days.

Despite the improvements, we observed a slight elevation in SA-β-Gal activity in resulting iMSCs relative to the JPCs, although the differences were not significantly different. Gene expression measurements of the senescence markers P16 and P21 over multiple passages confirmed our hypothesis that there is a correlation between iMSC maturation and their susceptibility to stress-induced senescence. This is most evident in the P21 gene expression analysis (Fig. 6B), where a distinct trend towards declining expression with increasing passages was observed. While the values of the iMSCs in P0 were 56.5% (-SB) and 33.1% (+ SB) higher than those of the JPCs, the values in P3 were 53.7% (-SB) and 54.9% (+ SB) lower than those of the JPCs. In terms of P16 expression, a similar trend was observed in the iMSC group treated with SB. Without the addition of SB, iMSCs showed higher P16 gene expression levels, which could indicate a slower or incomplete differentiation of the cells. In contrast to replicative senescence, which increases with passage and reduces the osteogenic potential of MSCs, the negative correlation between mineralization and senescence we observed here, both with increasing passage and in samples with and without SB, supports the hypothesis that senescence is a sign of incomplete maturation of iMSCs^51^.

Another significant improvement of the differentiation protocol was achieved by controlling the number of differentiated cells during passaging at the end of P0. As previously described, the dissociation of iPSCs results in apoptosis-mediated cell death. Conversely, MSCs remain unresponsive to dissociation. During the process of iMSC differentiation, particularly at passage 0, the cultures consist of a mixture of differentiated and undifferentiated cells. In order to select differentiated cells only, coatings and ROCK inhibitor were omitted during the first passaging on day 10. As a result, undifferentiated cells fail to adhere and undergo apoptosis and the differentiated cells adhere to the plate and survive. The optimal seeding density for a T75 flask is 1 million cells (13,333 cells/cm²), which should be maintained to ensure optimal differentiation. Given the considerable variation in the proportion of differentiated cells present in P0, there is a resulting variation of adhering cell numbers in P1 if the same number of cells is transferred to the next passage. However, the cell density in P1 is of relevance, since our observations have shown that an initially excessive confluency in P1 has the effect of inhibiting the mesenchymal differentiation of iPSCs. Conversely, a low confluency in P1 resulted in cell apoptosis, similar to what was observed in the dissociation of iPSCs. The quantification of differentiated cells via flow cytometry using the MSC marker CD105 when passaging cells from P0 to P1, enabled the control of cell density in P1 by passaging 1 million CD105^+^ cells, thereby achieving an improvement and standardization of the differentiation efficiency.

Taken together, we significantly enhanced the osteogenic potential of generated iMSCs by using SB431542, reduced premature senescence by avoiding iPSC separation and increased reliability and reproducibility of the method by controlling the number of CD105^+^ cells transferred in passage1. Furthermore, the differentiation method described here does not use xenogenic reagents or supplements, but instead uses human platelet lysate, which can be provided in GMP quality and approved for the production of cell-based therapies. In conjunction with our previously published method for iPSC generation using an integration-free, self-replicating RNA vector under xeno-free conditions, we present methods that can be readily adapted for the production of clinical-grade iMSCs^39^.

## Conclusion

In this study, we established an improved protocol to reliably yield iMSCs with high osteogenic potential. By avoiding the dissociation of iPSCs as well as measuring of CD105^+^ cells and thereby controlling cell density, stress-induced senescence caused by the mesenchymal differentiation process could be considerably reduced. Further, the addition of the TGF-β inhibitor SB431542 for the first five or ten days of iMSC differentiation yielded iMSCs with an enhanced osteogenic potential. Generated iMSCs needed at least 3 passages (approx. 25–30 days) for maturation until the osteogenic potential was comparable to that of original JPCs.

## Data Availability

The datasets used and analyzed during the current study are available from the corresponding author on reasonable request.
